# Supplementary material for: Modeling glioblastoma heterogeneity as a dynamic network of cell states
Source: Mol Syst Biol. 2021 Sep 16;17(9):e10105. doi: 10.15252/msb.202010105 (PMC8444284; doi:10.15252/msb.202010105)
Supplement: Supplementary file 6 — Source Data for Figure 5 [file MSB-17-e10105-s004.zip › Figure5A_sourcedata/GSEA_3071/nosigdb_stateVI.GseaPreranked.1622795169056/LARSSON_STATE_1.html]

Details for gene set LARSSON\_STATE\_1[GSEA]

|  || Dataset | state63071 |
| Phenotype | NoPhenotypeAvailable |
| Upregulated in class | na\_pos |
| GeneSet | LARSSON\_STATE\_1 |
| Enrichment Score (ES) | 0.46321234 |
| Normalized Enrichment Score (NES) | 3.0448112 |
| Nominal p-value | 0.0 |
| FDR q-value | 0.0 |
| FWER p-Value | 0.0 |
Table: GSEA Results Summary

  

Fig 1: Enrichment plot: LARSSON\_STATE\_1      
 Profile of the Running ES Score & Positions of GeneSet Members on the Rank Ordered List

  

| PROBE | GENE SYMBOL | GENE\_TITLE | RANK IN GENE LIST | RANK METRIC SCORE | RUNNING ES | CORE ENRICHMENT || 1 | HMGA1 |  |  | 1 | 1.500 | 0.0383 | Yes |
| 2 | DKK1 |  |  | 9 | 1.134 | 0.0655 | Yes |
| 3 | CAV1 |  |  | 13 | 1.087 | 0.0926 | Yes |
| 4 | DCBLD2 |  |  | 16 | 1.066 | 0.1195 | Yes |
| 5 | EIF6 |  |  | 28 | 0.969 | 0.1413 | Yes |
| 6 | ODC1 |  |  | 38 | 0.918 | 0.1624 | Yes |
| 7 | CAVIN1 |  |  | 44 | 0.888 | 0.1839 | Yes |
| 8 | COTL1 |  |  | 60 | 0.851 | 0.2016 | Yes |
| 9 | CAPN2 |  |  | 67 | 0.818 | 0.2209 | Yes |
| 10 | ANXA2 |  |  | 79 | 0.783 | 0.2380 | Yes |
| 11 | SMS |  |  | 88 | 0.761 | 0.2553 | Yes |
| 12 | C12orf75 |  |  | 99 | 0.738 | 0.2715 | Yes |
| 13 | PKM |  |  | 125 | 0.699 | 0.2825 | Yes |
| 14 | IGFBP5 |  |  | 145 | 0.671 | 0.2945 | Yes |
| 15 | ANK3 |  |  | 165 | 0.638 | 0.3055 | Yes |
| 16 | LDHA |  |  | 197 | 0.604 | 0.3124 | Yes |
| 17 | TUBA1C |  |  | 199 | 0.604 | 0.3277 | Yes |
| 18 | PLP2 |  |  | 205 | 0.600 | 0.3417 | Yes |
| 19 | TXN |  |  | 209 | 0.597 | 0.3562 | Yes |
| 20 | UPP1 |  |  | 218 | 0.590 | 0.3692 | Yes |
| 21 | PMAIP1 |  |  | 255 | 0.561 | 0.3735 | Yes |
| 22 | CLIC1 |  |  | 258 | 0.561 | 0.3874 | Yes |
| 23 | SH3BGRL3 |  |  | 265 | 0.556 | 0.4000 | Yes |
| 24 | SYNJ2 |  |  | 270 | 0.555 | 0.4132 | Yes |
| 25 | SRM |  |  | 292 | 0.544 | 0.4213 | Yes |
| 26 | LGALS1 |  |  | 323 | 0.526 | 0.4265 | Yes |
| 27 | USP53 |  |  | 340 | 0.515 | 0.4352 | Yes |
| 28 | PTTG1 |  |  | 344 | 0.514 | 0.4476 | Yes |
| 29 | PFN1 |  |  | 347 | 0.513 | 0.4602 | Yes |
| 30 | LMNA |  |  | 383 | 0.496 | 0.4632 | Yes |
| 31 | ACTG1 |  |  | 508 | 0.453 | 0.4402 | No |
| 32 | WDR1 |  |  | 540 | 0.444 | 0.4429 | No |
| 33 | CDC42EP3 |  |  | 545 | 0.443 | 0.4532 | No |
| 34 | FBXO32 |  |  | 556 | 0.438 | 0.4617 | No |
| 35 | ARL4C |  |  | 666 | 0.408 | 0.4417 | No |
| 36 | RPL8 |  |  | 728 | 0.391 | 0.4347 | No |
| 37 | PRELID1 |  |  | 760 | 0.384 | 0.4359 | No |
| 38 | PLIN3 |  |  | 781 | 0.380 | 0.4401 | No |
| 39 | TPI1 |  |  | 972 | 0.350 | 0.3959 | No |
| 40 | UBE2S |  |  | 974 | 0.349 | 0.4047 | No |
| 41 | PYGB |  |  | 993 | 0.347 | 0.4086 | No |
| 42 | CAST |  |  | 1070 | 0.334 | 0.3959 | No |
| 43 | GADD45GIP1 |  |  | 1154 | 0.323 | 0.3810 | No |
| 44 | S100A6 |  |  | 1193 | 0.317 | 0.3785 | No |
| 45 | RPS12 |  |  | 1219 | 0.313 | 0.3796 | No |
| 46 | ACTB |  |  | 1243 | 0.310 | 0.3811 | No |
| 47 | TMSB10 |  |  | 1289 | 0.305 | 0.3764 | No |
| 48 | TWIST1 |  |  | 1418 | 0.290 | 0.3481 | No |
| 49 | TMEM158 |  |  | 1466 | 0.283 | 0.3422 | No |
| 50 | DRAP1 |  |  | 1590 | 0.269 | 0.3147 | No |
| 51 | CTSC |  |  | 1767 | 0.254 | 0.2721 | No |
| 52 | GCHFR |  |  | 1820 | -0.252 | 0.2640 | No |
| 53 | STK17A |  |  | 2336 | -0.340 | 0.1288 | No |
| 54 | CTNNBIP1 |  |  | 2606 | -0.402 | 0.0639 | No |
| 55 | PDLIM4 |  |  | 2678 | -0.419 | 0.0548 | No |
| 56 | PMEPA1 |  |  | 3338 | -0.804 | -0.1087 | No |
| 57 | TMEFF2 |  |  | 3344 | -0.808 | -0.0893 | No |
| 58 | CSRP2 |  |  | 3484 | -1.081 | -0.1004 | No |
| 59 | DPYSL3 |  |  | 3521 | -1.188 | -0.0799 | No |
| 60 | SPP1 |  |  | 3541 | -1.286 | -0.0522 | No |
| 61 | GAP43 |  |  | 3568 | -1.438 | -0.0225 | No |
| 62 | IGFBP3 |  |  | 3588 | -1.623 | 0.0140 | No |
Table: GSEA details [plain text format]

  

Fig 2: LARSSON\_STATE\_1: Random ES distribution      
 Gene set null distribution of ES for **LARSSON\_STATE\_1**
